# Angiotensin-Converting Enzyme Inhibitor-Induced Angioedema With Resultant Airway Obstruction Following Endotracheal Intubation

**DOI:** 10.7759/cureus.37594

**Published:** 2023-04-14

**Authors:** Parsia Vazirnia, Yasmine Choroomi, Gifty Marfowaa, Abrahim N Razzak, Brian Quinn

**Affiliations:** 1 School of Medicine, Medical College of Wisconsin, Milwaukee, USA; 2 School of Medicine, Rosalind Franklin University of Medicine and Science, North Chicago, USA; 3 Internal Medicine, Medical College of Wisconsin, Milwaukee, USA

**Keywords:** endotracheal intubation complications, airway edema, intubation, tracheal stenosis, ace inhibitor induced angioedema

## Abstract

Angiotensin-converting enzyme inhibitor (ACEi)-induced angioedema is a consequence of excessive levels of bradykinin and accounts for nearly a third of angioedema cases when patients present to emergency rooms. While rare, patients can present with swelling in the face, tongue, and airways making it a life-threatening emergency. To secure an airway, endotracheal intubation may be conducted; however, tracheal stenosis is a known complication. In this report, we present a 61-year-old female with a history of ACEi-induced angioedema care in which she was intubated with facial swelling. Upon a repeat hospitalization, the patient developed stridor with respiratory distress. Bronchoscopy revealed severe tracheal stenosis with multilevel damage to tracheal rings, warranting urgent tracheostomy. One month after discharge, the patient was seen by an ENT specialist who performed a transnasal laryngoscopy revealing near total subglottic and tracheal stenosis of 3 cm stenosis length, presumed secondary to traumatic intubation for prior angioedema management. This case highlights the importance of careful intubation practices in patients with suspected airway edema.

## Introduction

Angiotensin-converting enzyme inhibitor (ACEi)-induced angioedema is a rare but life-threatening adverse effect of ACEi therapy with an incidence of approximately 0.1-0.7% [1]. There have been numerous instances in which patients with this clinical picture have required intubation or tracheostomy in severe cases [2-5]. This report presents a case of tracheal stenosis brought about by traumatic intubation. In the process of treating airway narrowing due to ACEi-induced angioedema, emergent intubation led to near-total subglottic stenosis. 

This case has been previously presented at the Society of Hospital Medicine Converge Scientific Meeting on April 7-10, 2022, at Nashville, Tennessee, and the American College of Physicians Wisconsin Chapter Scientific Meeting on September 9, 2022, at Wisconsin Dells, Wisconsin.

## Case presentation

**Figure 1:**
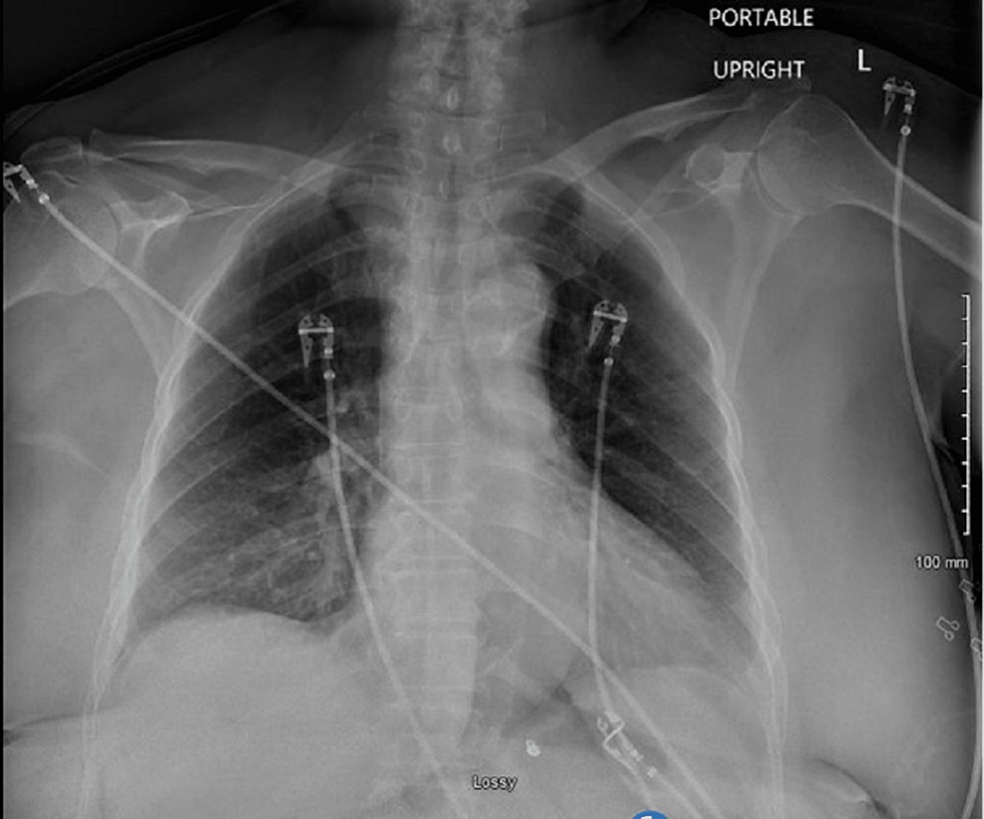
Initial chest radiograph scan showing no active signs of disease upon presentation

A 61-year-old female with a medical history significant for chronic pancreatitis status post pancreas shunt, obesity, hyperlipidemia, cerebrovascular accident over 20 years ago, hypertension, and type two diabetes, was previously admitted to the emergency department with angioedema and shortness of breath after ACEi use (lisinopril) for her hypertension. During this time, she underwent a difficult intubation due to diffuse facial swelling and throat tightness that led to airway compromise and respiratory failure. She exhibited metabolic derangements with an arterial blood gas test showing a pH of 7.52, partial pressure of oxygen (pO2) of 130 mmHg, partial pressure of carbon dioxide (pCO2) of 32 mmHg, base excess of 3.6 mmol/L, and a respiratory rate of 27 breaths per minute. Her metabolic panel also revealed a blood urea nitrogen level of 35mg/dL. The initial chest radiograph demonstrated no active signs of disease (Figure 1).

**Figure 2:**
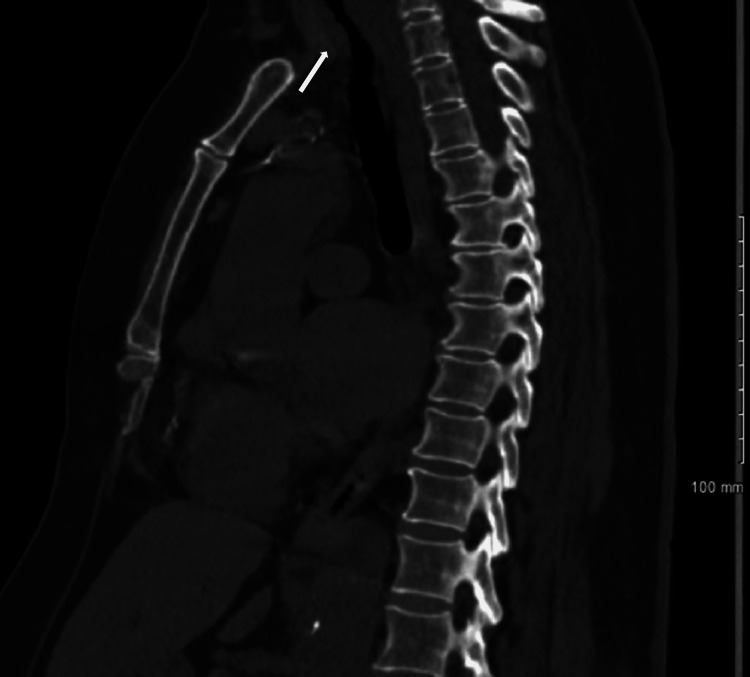
Sagittal view of computed tomography scan of chest without contrast showing tracheal stenosis

**Figure 3:**
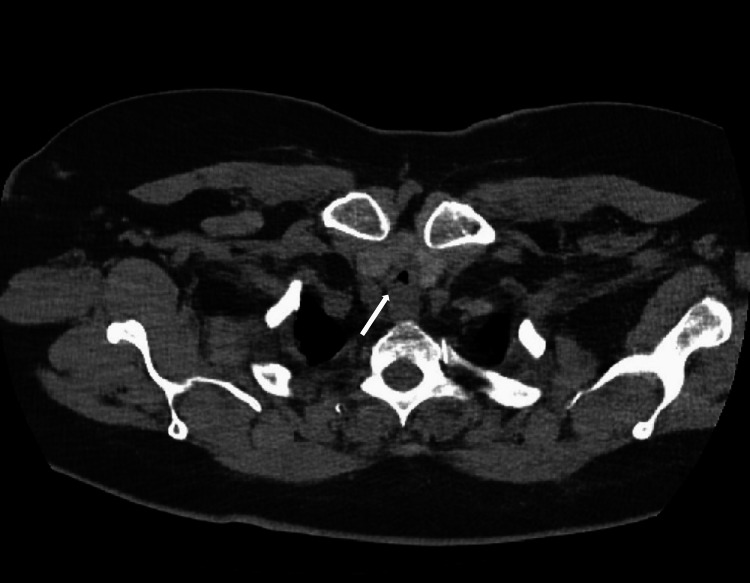
Axial view of computed tomography scan of chest without contrast showing subglottic concentric tracheal stenosis measuring 5.57 cm x 5.20 cm in dimension

**Figure 4:**
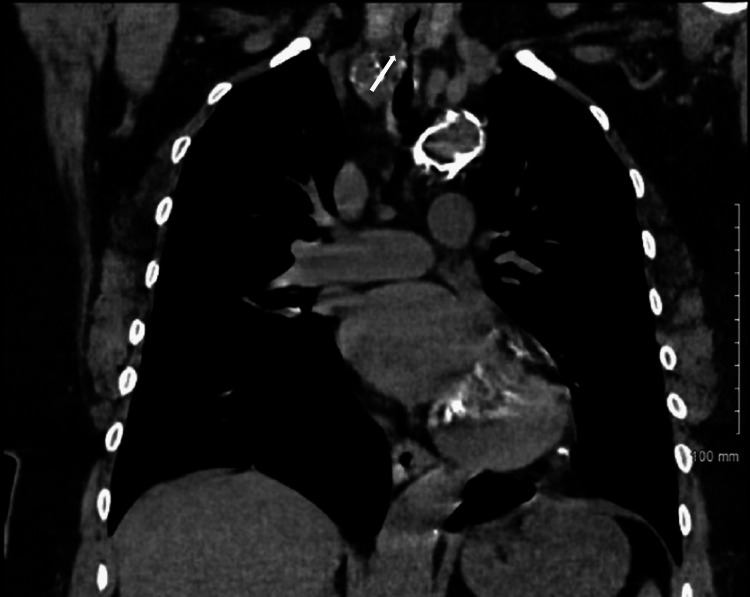
Coronal view of computed tomography scan of chest without contrast showing subglottic concentric tracheal stenosis measuring 2.6 cm in length

After a week of intubation and stabilizing her derangements, she was discharged; however, shortly after she was readmitted to the emergency department due to stridor and worsening shortness of breath with exertion. The patient was placed on 15L oxymask due to respiratory distress (weaned off to 2L) at which point she remained stable. ENT specialists’ laryngoscopy revealed an edematous larynx but a normal upper airway. However, interventional pulmonology unexpectedly found a severely narrowed and edematous airway with exposure to cartilaginous rings visualized by a subsequent flexible fiberoptic video bronchoscope introduced through the laryngeal mask airway. Starting just below the subglottis, there was severe tracheal stenosis with highly edematous mucosa and multilevel fractures to the tracheal rings. The extent of the stenosis can be seen on computed tomography imaging of the chest taken at clinical presentation (Figures 2-4).

Balloon dilation to 12 mm was gently performed using a 13-14-15 controlled radial expansion balloon catheter (Boston Scientific Corporation, Marlborough, Massachusetts, United States). ENT was contacted intraoperatively for consideration of urgent tracheostomy, and the case was discussed with interventional pulmonology, ENT, anesthesia, as well as the patient’s power of attorney. It was deemed unsafe for her to extubate, and the decision was made to proceed with tracheostomy. Intraoperative bronchoscopy guidance was provided during the open tracheostomy insertion. A 6.0 cuffed Shiley™ tracheostomy tube (Medtronic plc, Dublin, Ireland) was placed between the third and fourth rings and extended down to the fifth and sixth rings. Afterward, the patient reported improved breathing and was able to tolerate oral medication/nutrition without nausea or emesis. Prior to discharge, she developed acute hyperkalemia suspected to be due to trimethoprim-sulfamethoxazole use for *Pneumocystis jirovecii* prophylaxis. After discontinuation of this prophylaxis and use of sodium zirconium cyclosilicate (Lokelma), hyperkalemia resolved. The patient remained stable and was discharged to her home along with tracheostomy care education.

The patient’s tracheostomy tube was later replaced with a cuffless one to allow it to be more easily changed at home. One month after discharge, the patient was seen by an ENT specialist. She was aphonic at that appointment with plans for staging/exploratory direct microlaryngoscopy and bronchoscopy (with carbon dioxide laser) to determine the full extent of stenosis. There was also need to evaluate the possibility of establishing voicing with the tracheostomy tube. She later elected to undergo the procedure; an Ossoff-Pilling laryngoscope was placed into the oral cavity and into the endolarynx to evaluate the vocal cords. The patient was noted to have a markedly retroflexed and floppy epiglottis that made exposure of the endolarynx very challenging. Multiple attempts were made by staff, after which they were eventually able to visualize the endolarynx. The patient's larynx was reported to be relatively anterior which further complicated the procedure. With the scope, they were able to see the entire laryngeal appearance as well as the subglottis. There was marked pooling of secretions in the immediate subglottis with no visible tracheal connection between the subglottis and the distal airway. Tracheal stenosis was found above the tracheostomy tube, which measured 4 cm below the vocal cords. There was also evidence of mild granuloma of the right posterior false vocal cord. They were unable to do any effective dilation and the patient continued to use a 6.0 cuffless tracheostomy tube.

Due to the severity of her situation, she was referred to an outside institution for an opinion on surgical options. She was seen at the outside institution for lysis of tracheal scar/recanalization as well as T-tube trimming and replacement. The patient is on a current plan to leave the newest tracheostomy tube in place for at least six months for adequate healing. She is reportedly doing well but still has no significant voicing. 

## Discussion

ACEi-induced angioedema has an incidence of 0.1-0.7% and is higher in women and African Americans (presumably due to polymorphism in the gene encoding aminopeptide P that is responsible for the metabolism of ACEi). It accounts for approximately one-third of angioedema cases in the emergency department [2,6-8]. Mechanistically, ACEi-induced angioedema is a consequence of excessive levels of bradykinin [6,9-12]. 

Angioedema associated with ACEi presents with swelling in the face, tongue, and airways [2,13]. Symptoms can occur within a week to years after ACEi treatment [3,14]. This condition warrants emergency intervention as it can lead to life-threatening obstruction of airways [2,3,13,14]. The preferred management for ACEi-induced angioedema depends on the clinical picture, as there are no laboratory studies available currently to establish this particular etiology and diagnosis compared to hereditary angioedema, which is diagnosed with low levels of C2, C4, and C1 esterase inhibitors [4]. While a case of angioedema presenting with lip and soft palate edema will be treated more conservatively, a more severe case accompanied by lingual and laryngeal swelling will require intensive care unit admission with a cricothyrotomy and tracheostomy kit near the bedside [15]. In some cases, however, surgical stress and tracheal intubation itself have been associated with the acute onset of an episode of ACEi-induced angioedema [2]. Regardless of the etiology of the episode of angioedema, there is a significant risk to the patient and around 25% of patients will ultimately require intubation for airway protection [5]. As intubation has become common for airway protection in angioedema patients, it is important to note that it can come with risks as well. Therefore, providers must take extreme caution during the intubation as subsequent airway trauma may occur [3]. 

One rare but major complication of tracheal intubation and tracheostomy is post-intubation tracheal stenosis, yet the mechanism for intubation-induced tracheal stenosis is not fully understood [3,16-18]. The underlying pathology of tracheal stenosis is thought to be attributed to cartilage ulceration and inflammatory reactions, leading to granulation and fibrous tissue formation, which ultimately causes stenosis [19]. Additionally, ischemia from direct contact with the endotracheal tube or via increased pressure with the cuff of the tube has been considered an underlying cause of this complication [19]. Tracheal intubation accounts for 19% of cases of iatrogenic subglottic tracheal stenosis while tracheostomies account for 65% of cases [16]. Furthermore, a higher incidence of this complication is found in patients who have keloids, since the abnormal proliferation of connective tissue in response to trauma or injury can lead to tracheal stenosis [16]. Tracheal stenosis can be repaired operatively, but successful results have also been obtained in a less invasive manner through balloon bronchoplasty [3,19]. Bronchoscopy balloon dilatation using an angioplasty balloon catheter was found to be most successful in children and select cases with adults. When the balloon is placed in the right location, it has a rapid expansive force on the stenotic location; this evenly distributes the force over the circumference of the stenosis while simultaneously minimizing the risk of airway rupture. The surgeon also has more flexibility and control of the force of the balloon, making this an advantageous procedure compared to alternative dilatation instruments [19]. In instances where tracheal stenosis is unresponsive to balloon dilation (which is a more conservative treatment option), tracheal resection is performed; resection is reported to have a success rate of 71-95% [20]. However, contraindications to tracheal resection include tracheal stenosis that requires resection of over half the trachea; this may create additional tension upon closure. Given our patient's near-total subglottic tracheal stenosis as well as her various comorbidities, tracheal resection may not have been a suitable treatment option requiring referral to an outside institution for a second opinion. 

## Conclusions

This report summarizes an unusual instance of tracheal stenosis secondary to intubation during ACEi-induced angioedema eventually requiring tracheostomy. This case highlights the importance of careful intubation practices in patients with known or suspected airway edema and consideration of endotracheal stenosis in the differential diagnosis of patients with a history of angioedema necessitating intubation.
